# Anti-Mycobacterial Evaluation of 7-Chloro-4-Aminoquinolines and Hologram Quantitative Structure–Activity Relationship (HQSAR) Modeling of Amino–Imino Tautomers

**DOI:** 10.3390/ph10020052

**Published:** 2017-06-09

**Authors:** Marcelle L. F. Bispo, Camilo H. S. Lima, Laura N. F. Cardoso, André L. P. Candéa, Flávio A. F. M. Bezerra, Maria C. S. Lourenço, Maria G. M. O. Henriques, Ricardo B. Alencastro, Carlos R. Kaiser, Marcus V. N. Souza, Magaly G. Albuquerque

**Affiliations:** 1 Departamento de Química, Universidade Estadual de Londrina (UEL), Londrina 86057-970, Brazil; mlfbispo@uel.br; 2 Faculdade de Farmácia, Laboratório de Química Medicinal (LQMed), Programa de Pós-Graduação em Ciências Aplicadas a Produtos para Saúde, Universidade Federal Fluminense (UFF), Niterói 24241-000, Brazil; camilolimaster@gmail.com; 3 Programa de Pós-Graduação em Química (PGQu), Instituto de Química (IQ), Universidade Federal do Rio de Janeiro (UFRJ), Rio de Janeiro 21949-900, Brazil; lauranfc@hotmail.com (L.N.F.C.); bicca@iq.ufrj.br (R.B.A.); kaiser@iq.ufrj.br (C.R.K.); 4 Fundação Oswaldo Cruz (FioCruz), Instituto de Tecnologia em Fármacos (Far-Manguinhos), Rio de Janeiro 21041-250, Brazil; andrelpc@far.fiocruz.br (A.L.P.C.); gracahen@far.fiocruz.br (M.G.M.O.H.); 5 Fundação Oswaldo Cruz (FioCruz), Instituto de Pesquisas Clínicas Evandro Chagas (IPEC), Rio de Janeiro 21040-360, Brazil; flavio.ferreira@ipec.fiocruz.br (F.A.F.M.B.); cristina.lourenco@ini.fiocruz.br (M.C.S.L.)

**Keywords:** *Mycobacterium tuberculosis*, 4-aminoquinoline, amino–imino tautomerism, molecular modeling, hologram quantitative structure–activity relationship (HQSAR), drug design, anti-mycobacterial activity, cytotoxicity

## Abstract

In an ongoing research program for the development of new anti-tuberculosis drugs, we synthesized three series (**A**, **B**, and **C**) of 7-chloro-4-aminoquinolines, which were evaluated in vitro against *Mycobacterium tuberculosis* (MTB). Now, we report the anti-MTB and cytotoxicity evaluations of a new series, **D** (**D01**–**D21**). Considering the active compounds of series **A** (**A01**–**A13**), **B** (**B01**–**B13**), **C** (**C01**–**C07**), and **D** (**D01**–**D09**), we compose a data set of 42 compounds and carried out hologram quantitative structure–activity relationship (HQSAR) analysis. The amino–imino tautomerism of the 4-aminoquinoline moiety was considered using both amino (I) and imino (II) forms as independent datasets. The best HQSAR model from each dataset was internally validated and both models showed significant statistical indexes. Tautomer I model: leave-one-out (LOO) cross-validated correlation coefficient (q^2^) = 0.80, squared correlation coefficient (r^2^) = 0.97, standard error (SE) = 0.12, cross-validated standard error (SE_cv_) = 0.32. Tautomer II model: q^2^ = 0.77, r^2^ = 0.98, SE = 0.10, SE_cv_ = 0.35. Both models were externally validated by predicting the activity values of the corresponding test set, and the tautomer II model, which showed the best external prediction performance, was used to predict the biological activity responses of the compounds that were not evaluated in the anti-MTB trials due to poor solubility, pointing out **D21** for further solubility studies to attempt to determine its actual biological activity.

## 1. Introduction

Tuberculosis (TB), a severe infectious bacterial disease caused by *Mycobacterium tuberculosis* (MTB), infected almost 9.6 million of people around the world and caused 1.5 million deaths in 2014 [1]. In the last five years, TB drug-resistant cases have increased dramatically; the number of multidrug-resistant (MDR) TB (i.e., resistance to at least both isoniazid and rifampicin) reported cases has almost doubled (250,000 to 440,000), while extensively drug-resistant (XDR) TB (i.e., resistance to any fluoroquinolone, and at least one of three second-line injectable drugs, i.e., capreomycin, kanamycin, and amikacin, in addition to MDR) was identified in 105 countries in 2015 [1].

Considering the alarming global situation of TB, as well as the high impact of MDR/XDR-TB on TB treatment, there is an urgent need to develop new anti-TB drugs. In this context, the quinoline nucleus, which occurs in natural products, besides being used in the design of bioactive compounds, is considered a “privileged structure” [2], showing a variety of biological activities, e.g., anticancer [3], antibacterial [4], antimalarial [5], antileishmanial [6], and anti-viral (e.g., anti-human immunodeficiency virus, HIV) [7].

In relation to anti-MTB activity, in December 2012, the United States Food and Drug Administration (U.S. FDA) approved a diarylquinoline derivative, namely bedaquiline (Sirturo® by Janssen/Johnson & Johnson), showing a new mechanism of action [8]. Bedaquiline has shown activity against MTB with minimum inhibitory concentration (MIC) values for the MTB wild-type H37Rv strain (MIC ≤ 0.1 µM) as well as MDR strains (MIC ≤ 0.5 µM) [9].

**Figure 1:**
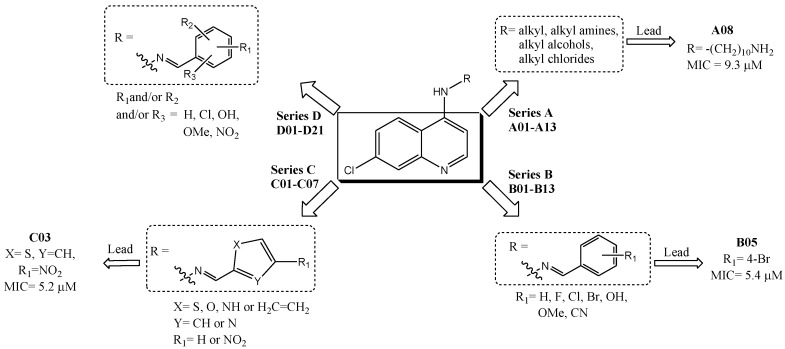
Series **A**–**D** of 7-chloro-4-aminoquinoline derivatives synthesized and evaluated against the *Mycobacterium tuberculosis* wild-type H37Rv strain (minimum inhibitory concentration, MIC) by our research group and their respective lead compounds.

Based on these observations and on our continuing research program for the development of new anti-MTB agents, [10,11,12,13,14,15] we reported the synthesis and anti-mycobacterial activity of three different series of 7-chloro-quinoline (7CQ) derivatives with promising activities, namely, *N*-alkyl-substituted 4-aminoquinolines (**A01**–**A13**) [11], monosubstituted phenyl 4-quinolinyl-hydrazones (**B01**–**B13**) [10], and 4-heteroaromatic quinolinyl-hydrazones (**C01**–**C07**) [12]. Among them, three compounds (**A08**, **B05**, and **C03**) showed significant biological activity, when compared with the ethambutol (EMB) standard drug, and they could be considered interesting lead compounds in the development of new anti-TB drugs (Figure 1).

Among series **A**, **B**, and **C**, series **B** showed the most promising pharmacological and toxicological profiles [10]. Hence, in this article, we evaluate the influence of two or more substituent groups on the phenyl ring, based on the different stereoelectronic characteristics of these groups. For this reason, in this current work, we report the biological evaluation of series **D** against the MTB wild-type H37Rv strain (Figure 1, Table 1), composed of polysubstituted phenyl 7-chloro-4-quinolinyl-hydrazones (**D01**–**D21**) previously synthesized by our research group [3].

Furthermore, this series promotes an increase in the structural diversity allowing the utilization of the ligand-based strategy analysis using the hologram quantitative structure–activity relationship (HQSAR) method [16] with the purpose of driving the structural modification to improve the anti-mycobacterial (anti-MTB) activity of these series of compounds.

In the HQSAR method, the occurrence of each 2D molecular fragment derived from each molecule in the dataset forms a set of molecular (hologram or fingerprint) descriptors (X, independent variables), which are correlated to the biological activity response (Y, dependent variable) [16,17,18,19].

In addition, this method avoids the need for molecular alignment or knowledge of bioactive conformation inherent in the current 3D-QSAR methods, such as the Comparative Molecular Field Analysis (CoMFA) [20], and is a good choice for datasets lacking a defined biological target.

## 2. Results and Discussion

### 2.1. Biological Activities

The synthesis, purification and spectroscopic data of polysubstituted 7-chloro-4-quinolinyl-hydrazone derivatives (**D01**–**D21**) were previously reported by our research group [3]. The antimycobacterial activities of **D01**–**D21** were evaluated against the *M. tuberculosis* H37Rv strain susceptible to streptomycin, isoniazid, rifampin, and ethambutol (S.I.R.E.) drugs (American Type Culture Collection, ATCC^®^ 27294™), using the microplate Alamar blue assay (MABA) [21,22], and the results are expressed in terms of minimum inhibitory concentration (MIC, µM) (Table 1).

According to Table 1, nine derivatives (**D01**–**D09**) showed moderate activity against *M. tuberculosis*, among them, **D05** (MIC = 7.3 μM), **D06** (MIC = 7.3 μM), **D07** (MIC = 18.3 μM), **D08** (MIC = 16.8 μM), and **D09** (MIC = 17.3 μM) showed anti-MTB activity comparable to the standard drug (EMB, MIC = 15.3 μM) used in the MABA assay.

However, compounds **D10**–**D21** could not be evaluated by this method due to their low solubility in the culture medium, which may be related to a change in molecular polarity when more substituents were added on the phenyl ring, or physical factors, such as crystalline form, which may influence the dissolution rates in the Middlebrook 7H9 medium [23,24].

Moreover, the cytotoxicity profile of the active compounds (**D01**–**D09**) were also evaluated in the presence and absence of each compound by the Mosmann’s MTT [methylthiazoletetrazolium bromide, i.e., 3-(4,5-dimethyl-1,3-thiazol-2-yl)-2,5-dimethyl-2H-tetrazol-3-ium bromide Merck] assay [25,26], and it is expressed as a percentage of cellular viability (Table 1). According to Table 1, **D01** (cell viability = 100%), **D04** (cell viability = 100%), and **D05** (cell viability = 98%) do not kill more than 5% of cells at the lowest tested concentration (2.5 μg/mL), suggesting that non-cytotoxic profile is maintained for these compounds when compared to series **B**.

### 2.2. HQSAR (2D-QSAR) Modeling

In order to rationalize the structure–activity relationship (SAR) of the active derivatives from series **D** (**D01**-**D09**) and to guide future structural modifications, these compounds and 33 more from series **A** (**A01**–**A13**) [11], **B** (**B01**–**B13**) [10], and **C** (**C01**–**C07**) [12] were used to construct robust HQSAR models, considering both amino (I) and imino (II) tautomers as independent datasets (Table 2), since this potential amino–imino tautomerism could be important in the microenvironment of the target receptor. The 42 compounds divided into training set (32 compounds) and test set (10 compounds) present a regular distribution over the entire range of experimental pMIC (–Log of the minimum inhibitory concentration) values.

Initially, the 12 default hologram lengths (HL) were tested, aiming to reduce the chance of bad collisions. Then, the default fragment size (FS) (4–7 atoms) was used, setting the maximum number of components (NC) to fifteen, which is smaller than half the number of compounds used in the training set (32 compounds). Finally, the HQSAR runs were performed varying the six types of fragment distinction (FD) parameter (i.e., A = atoms, B = bonds, C = connections, Ch = chirality, H = hydrogen atoms, DA = hydrogen bond donor/acceptor atoms), obtaining twelve different models (Table 3).

The three most predictive HQSAR models from this first optimization step of the tautomer I dataset (models A/C, A/C/H, and C/DA, Table 3) showed comparable leave-one-out (LOO) cross-validated r^2^ (q^2^) values. In the first model, the atoms (A) and connections (C) FD parameters are combined: A/C (q^2^= 0.80; cross-validated standard error, SE_cv_ = 0.32). The second model combines the atoms (A), connections (C), and hydrogen atoms (H) FD parameters: A/C/H (q^2^ = 0.79; SE_cv_ = 0.33). In the third model, the connections (C) and donor/acceptor atoms (DA) FD parameters are combined: C/DA (q^2^ = 0.77; SE_cv_ = 0.39). However, the A/C model was selected because of the high q^2^ value, the low SE_cv_ value, and the lower NC and HL. For the tautomer II data set, the most predictive model obtained showed the same combination of FD parameters: atoms and connections (A/C, q^2^ = 0.77; SE_cv_= 0.35).

Aiming to improve the A/C models previously described for both tautomers I and II datasets, eight new models were generated considering FS starting from 2 to 5 until reaching 9 to 12 atoms per fragment, varying each fragment by four units. According to Table 4, the variation of this parameter did not improve the q^2^ value, which proved that the default FS (4–7 atoms) led to better statistical results compared to other fragment sizes.

In order to analyze the robustness of the obtained models, the Y-randomization test was performed ten times. In this test, the dependent variable (i.e., Y column, the biological activity data) was randomized and the HQSAR method was carried out for each training set (I and II). According to Table 5, the 10 randomized biological datasets are not correlated with the order of the original dataset (simple correlation coefficient, R, values close to zero); among the 10 runs, only 4 generated predictive models with low q^2^ and r^2^ values, indicating that an acceptable model was obtained for the original data set (i.e., non-randomized).

After the internal validation (i.e., leave-one-out cross-validation, LOO-cv) of the models derived from the I and II data sets, the ability of these models to predict the activity values of new compounds (i.e., the test set) was measured. Therefore, the external validation of the HQSAR models was performed by predicting the biological potencies of the test set compounds using the best model derived from each training set. The predictive ability of the HQSAR models is expressed with predictive r^2^ (r^2^_Pred_) values, which is similar to the cross-validated r^2^ (q^2^) and is defined by Equation (1) (see HQSAR Modeling in the Materials and Methods section).

**Figure 2:**
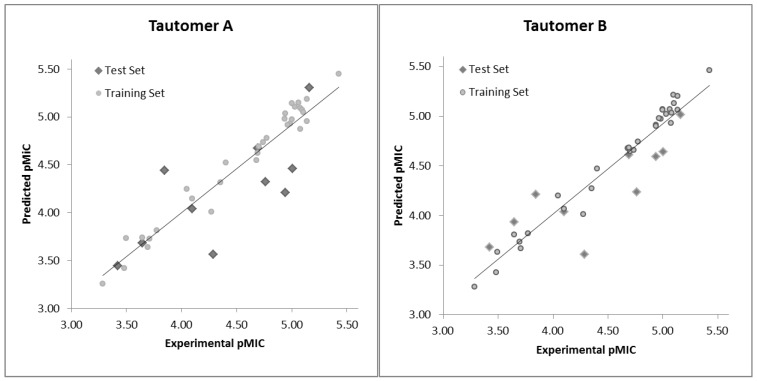
Plot of the experimental versus predicted pMIC values of the training and test sets of the tautomers I (amino) (A) and II (imino) (B) datasets.

The experimental pMIC (pMIC_Exp_) and calculated pMIC (pMIC_Calc_) values and residuals (pMIC_Exp_ – pMIC_Calc_, respectively) of the training and test sets compounds obtained by the best HQSAR model of each data set are reported in Table 6, and the comparison plots between the experimental and calculated potencies of both the training and test sets are shown in Figure 2.

The limitation of the predictive ability of the best models for the tautomers I and II datasets was determined by the presence of outliers, i.e., compounds of the training and test sets showing residual values exceeding twice the standard deviation (SD) of estimate of the model (tautomer I, 2 * SD = 0.47; tautomer II, 2 * SD = 0.38) [17,18,27].

According to this criterion, there are four outliers for the tautomer I model (**A01**, **B02**, **C02**, and **D01**) and only two outliers for the tautomer II model (**A01** and **D09**), all of them belonging to the test set (compounds marked with an asterisk in Table 6), but not exceeding one log unit (Table 6 and Figure 2). Although the tautomer I model (amino form) shows better statistical parameters for internal prediction (training set), it owns poor reliability on the external prediction (test set). Therefore, only the HQSAR tautomer II model (imino form) was used for the analysis of the HQSAR contribution maps to the biological activity of this dataset, as well for the prediction of the biological activity of the **A10**–**A21** derivatives.

**Figure 3:**
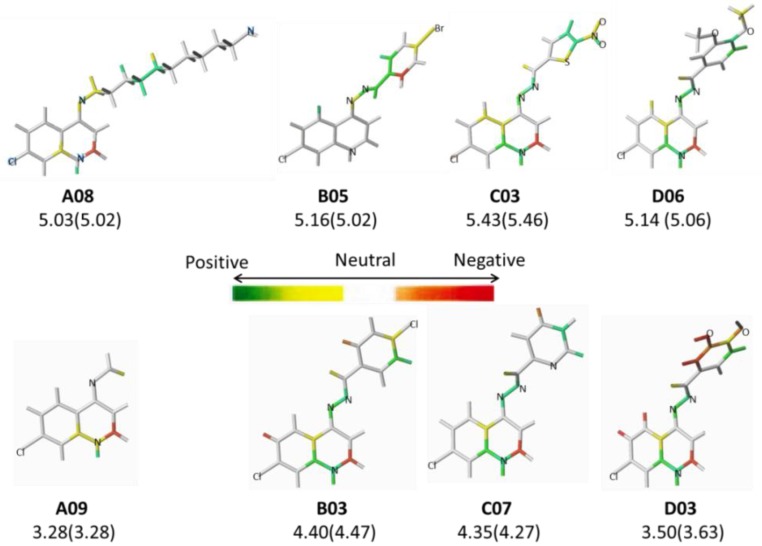
The HQSAR contribution maps of the most (**A08**, **B05**, **C03**, and **D06**) and least (**A09**, **B03**, **C07**, and **D03**) potent compounds according to the tautomer II model from each series (**A**, **B**, **C**, and **D**) along with the experimental (and calculated) pMIC values.

The SAR of the HQSAR models can be graphically represented by the contribution maps, where each compound can be colored using a standard color code that discriminates the individual atomic and connection contributions to the biological activity response: yellow, blue-green, and green reflect positive contribution; orange, orange-red, and red reflect negative contribution; and white reflects neutral contribution. Therefore, in order to understand the SAR of the HQSAR tautomer II model, the contribution maps of the most (**A08**, **B05**, **C07**, and **D06**) and least (**A09**, **B03**, **C03**, and **D03**) potent anti-MTB derivatives from each series (**A**, **B**, **C**, and **D**) are presented in Figure 3.

In the *N*-alkyl series (**A01**–**A13**), the contribution maps of **A08** (most potent) and **A09** (least potent) (Figure 3) show the presence of a long aliphatic chain in **A08**, as compared to a short chain in **A09**, which is related to a larger number of fragments increasing the potency. This effect may be related to a greater lipophilicity conferred by the longer aliphatic chain. Consequently, the increase in the number of methylene groups of the aliphatic chain has a positive contribution to the overall biological activity.

In the monosubstituted phenyl-hydrazone series (**B01**–**B13**), the contribution maps of **B05** (most potent) and **B03** (least potent) (Figure 3) show the substitution of chlorine (**B03**) by bromine (**B05**) in the *para* position of the phenyl ring, which is related to a larger number of fragments in phenyl-hydrazone subunit, increasing the activity. Again, this effect may also be related to a greater lipophilicity conferred by bromine. However, at the same time, this substitution almost changed the entire character of the quinoline ring, which has a neutral contribution in **B05**.

Analogous to series **B**, in the polysubstituted phenyl-hydrazone series (**D01**–**D21**), the contribution maps of **D06** (most potent) and **D03** (least potent) (Figure 3) show the substitution of the hydroxyl (**D03**) by methoxy (**D06**) group at both the *meta* and *para* positions of the phenyl ring, which is related to a larger number of fragments increasing the activity, especially in the phenyl ring and C5/C6 atoms of the quinoline ring. As in series **B**, this effect should be related to a greater lipophilicity conferred by the methoxy group. In fact, the bromine and methoxy group increases the lipophilicity of **B05** and **D06**, respectively, resulting in increased anti-MTB activity (Table 2).

Finally, in the heteroaromatic series (**C01**–**C07**), the contribution maps of **C03** (most potent) and **C07** (least potent) (Figure 3) show the replacement of the 2-pyridinyl by 5-nitro-2-thienyl group, which is related to a larger number of fragments increasing the activity, especially in both the heteroaromatic and quinoline rings. Again, this effect may be related to a greater lipophilicity conferred by the 5-nitro-2-thienyl group. On the other hand, heteroaromatic nitro compounds, such as **C03**, which is the most active of the dataset, are known for producing radical species that cause damage to bacterial DNA [28].

**Figure 4:**
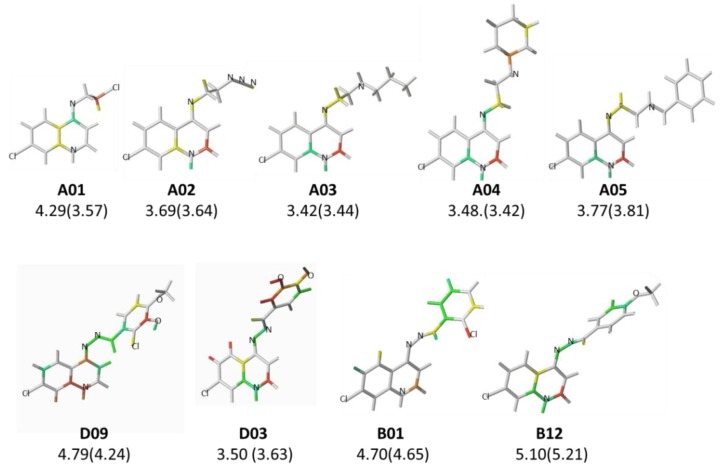
The HQSAR contribution maps of the outliers (**A01** and **D09**) and similar compounds that belong to the training set.

As previously stated, there are only two outliers in the HQSAR tautomer II model (Table 6 and Figure 2), and for both compounds, the pMIC values were underestimated (residuals of **A01** = 0.68 and **D09** = 0.53). The HQSAR model II underestimated the predictive pMIC value of outlier **A01**, besides, its experimental pMIC value is similar to those of compounds **A02**–**A05** (Figure 4). This behavior could be attributed to the fact that the exchange of the chlorine atom by bulky amines could influence the permeability of these compounds through the cell wall.

In relation to outlier **D09**, which have three different substituents attached to the phenyl ring, there is no similar compound in the training set, however, the HQSAR models were obtained with compounds showing substituents in the same positions found in this outlier, e.g. **D03**, **B01**, and **B12** (Figure 4). When comparing the contribution maps of these compounds, the presence of a hydroxyl substituent increases the number of unfavorable fragments, contributing to the low estimated value.

The HQSAR model II was used to predict the biological activities (pMIC_Calc_) of compounds with poor solubility in the culture media (**D10**–**D21**), where compounds with identical substituents (**D10**–**D15**) showed close pMIC_Calc_ values in comparison with those used in the training set (Table 7).

In the case of derivatives **D16**–**D21**, the HQSAR model II showed pMIC_Calc_ values which were not reliable due to the lack of compounds in the training test with substituents attached to the phenyl ring showing comparable chemical characteristics. Therefore, thermodynamic and kinetic studies are needed to determine their dissolution rates, according to the crystalline forms, to allow assessing their activities values [23,24]. This study would be especially interesting for compound **D21** since the presence of the nitro group in its structure resulted in increasing favorable fragments and the predicted activity value was greater than that of the most active compound in the data set.

## 3. Materials and Methods

### 3.1. General Procedures for Biological Tests

#### 3.1.1. Anti-Mycobacterial (Anti-MTB) Activity

The anti-MTB activities were tested using the microplate Alamar blue assay (MABA) [21,22]. Briefly, 200 µL of sterile deionized water was added to all outer-perimeter wells of sterile 96-well Falcon-3072 plates (Becton Dickinson, Lincoln Park, NJ, USA) to minimize evaporation of the medium in the test wells during incubation. The 96 plates received 100µL of the Middlebrook 7H9 broth (Difco Laboratories, Detroit, MI, USA), containing cultures of the *Mycobacterium tuberculosis* wild-type H37Rv strain susceptible to streptomycin, isoniazid, rifampin, and ethambutol (S.I.R.E.) drugs (American Type Culture Collection, ATCC^®^ 27294™), and a serial dilution of compounds D01–D21 was made directly on the plate. The final drug concentration ranged from 0.01 to 100 µg/mL. Plates were covered and sealed with parafilm and incubated at 37 °C for five days. After this time, 25 µL of a freshly prepared 1:1 mixture of Alamar blue (AccuMed International, Westlake, OH, USA) reagent and 10% Tween-80 was added to the plate and incubated for 24 h. A blue color in the well was interpreted as no bacterial growth, and a pink color was scored as growth. The minimal inhibitory concentration (MIC) was defined as the lowest drug concentration, which prevented a color change from blue to pink.

#### 3.1.2. Cytotoxicity Activity (Colorimetric Cell Viability Assay)

The cellular viability for a macrophage cell line J774 (ATCC^®^ TIB-67™) was determined by Mosmann’s MTT microculture assay [25,26]. Macrophage viability was evaluated in the presence and absence of test compounds (D01-D09). The cells were plated in flat bottom 96-well plates (2.5 × 10^6^ cells/well/100 µL) cultured for 24 h in a controlled atmosphere (CO_2_ 5% at 37 °C), and non-adherent cells were washed by gentle flushing with Roswell Park Memorial Institute (RPMI) 1640 medium, supplemented with fetal bovine serum (10%) and gentamicin (25 µg/mL). Adherent cells were infected or not with *Bacillus Calmette-Guérin* (BCG) (2.5 × 10^6^ colony-forming units (CFU)/well/100 µL) cultured in the presence of medium alone, Tween-20 (3%) (live and dead controls, respectively) or different concentrations of compounds (1.0, 10.0, and 100 µg/mL) in a triplicate assay. After 48 h, MTT stock solution (5 mg/mL of saline; 20 mL/well) was added to the culture and, 4 h later, the plate was centrifuged for 2 min at 2800 rpm, supernatant was discharged, dimethyl sulfoxide (DMSO) (100 µL/well) was added to solubilize the formazan crystals, and the absorbance was read at 540 nm in a plate reader (BioRad-450).

### 3.2. Computational Analysis

#### 3.2.1. Chemical and Biological Dataset

The dataset is composed of 42 compounds from series **A** (**A01**–**A13**) [11], **B** (**B01**–**B13**) [10], **C** (**C01**–**C07**) [12], and **D** (**D01**–**D09**). For series **A**, **B**, and **C**, the biological activity responses (i.e., minimum inhibitory concentration, MIC) were collected from the literature, [10,11,12] and for series **D**, from the current work (Table 1). The MIC (μM) values were expressed in negative logarithmic scale, i.e., pMIC (–LogMIC). The chemical structures and the corresponding pMIC (Molar, M) values for the entire dataset (**A01**–**A13**, **B01**–**B13**, **C01**–**C07** and **D01**–**D09**) are listed in Table 2.

For the HQSAR analysis, the dataset (Table 2) was divided into a training set (N_Training_ = 32 compounds) and a test set (N_Test_ = 10 compounds). In selecting the compounds to compose the training and test sets, the following steps were considered to ensure the same chemical and biological diversity in both sets: (i) a plot of experimental pMIC (pMIC_Exp_) versus experimental pMIC (pMIC_Exp_) values was generated to evaluate the distribution of the biological activity values; (ii) ten compounds (about 20 to 25% of total compounds, N_Total_ = 42) were randomly selected to compose the test set, excluding the most and least active compounds, but maintaining a regular distribution in the plot; (iii) the chemical structures of these 10 compounds were analyzed, checking if they were represented in the training set. Importantly, the test set compounds were not included during the steps of construction and selection of the best models, being used only in the external validation step.

The 42 compounds were built considering both tautomeric forms of the 4-aminoquinoline group, i.e., amino (tautomer I) and imino (tautomer II) forms, generating two datasets (Table 2). Such amino–imino tautomerism, which could be important in the microenvironment of the target receptor, is already well described in the literature, where the amino tautomer is generally favored. Recently, however, our group showed the coexistence of both amino and imino tautomers in a ratio of 1:1 in the crystal of (*E*)-N-7-chloro-4-(2-methoxy-cinnamoyl-hydrazinyl)quinoline recrystallized from 2-methoxyethanol solutions revealed by X-ray diffraction [29].

#### 3.2.2. Molecular Modeling

The chemical structures of these 42 quinoline derivatives, considering both amino (I) and imino (II) tautomers (Table 2), were constructed based on the crystallographic data of aryl aldehyde 7-chloro-quinoline-4-hydrazones from our group (CCDC n° 782421–782424) [30], available on the Cambridge Crystallographic Data Centre (CCDC) [31], using Spartan software (version 10) (Wavefunction, Inc.) [32]. All structures (amino and imino tautomers) were submitted to a systematic conformational analysis, using the Austin Model 1 (AM1) semi-empirical method, available in Spartan [33]. The most stable conformation of each amino and imino tautomer was submitted to a single-point calculation, using the Density Functional Theory (DFT)/Becke, 3-parameter, Lee-Yang-Parr (B3LYP) method with the 6-31+G(d) basis set, available in Spartan.

#### 3.2.3. HQSAR Modeling

HQSAR modeling was performed using the Sybyl software (version 8.0) [34]. During hologram generation, the HQSAR models may be affected by the hologram length (HL), fragment size (FS), and fragment distinction (FD) parameters. Then, different combinations of these parameters were considered in the HQSAR runs: the 12 default hologram lengths (53, 59, 61, 71, 83, 97, 151, 199, 257, 307, 353, and 401 bins), initially at the default fragment size (4–7 atoms) and then ranging from 2 to 12 atoms per fragment; and the six types of FD parameter, i.e., atoms (A), bonds (B), connections (C), hydrogen (H) atoms, chirality (Ch), and hydrogen bond donor/acceptor (DA) atoms.

The HQSAR models are generated using the partial least squares (PLS) analysis, while the internal validation (i.e., cross-validation) procedure is performed by the leave-one-out (LOO) approach, in order to determine the optimal number of components (NC) that yields the most predictive models. Therefore, the best HQSAR models were selected based on various statistical parameters, including the squared correlation coefficient (r^2^) and the LOO cross-validated r^2^ (q^2^) values.

In order to assess the risk of chance correlation [35], the Y-randomization (Y-scrambling or response randomization) test [36] was carried out, which is an additional validation procedure, by scrambling the original biological activity values and re-running the whole HQSAR analysis for the training set. The randomization of the biological activity (experimental pMIC) values was performed with a Python 2.7 script, developed in our laboratory, which randomly exchanges the order of the original data, then calculates the simple correlation coefficient (R) between the random order and the original order of the data.

In addition, an external validation was performed using the test set compounds, which were not considered for HQSAR model development. The predictive ability of the models was investigated by the predictive r^2^ (r^2^pred) values, defined according to Equation (1).

(1) $$r_{pred}^{2}=\frac{SD-PRESS}{SD}$$

In Equation (1), SD is the sum of squared deviations between the biological activity of the test set and the mean activity of the training set molecules, and PRESS (predictive residual error sum of the squares) is the sum of the squared deviations between the observed (experimental) and the predicted (calculated) activity values for every molecule in the test set [37]. Besides, all HQSAR procedure discussed above was applied to both datasets (4-aminoquinoline tautomers I and II data sets).

## 4. Conclusions

In this article, 21 polysubstituted 7-chloro-4-quinolinyl-hydrazone (7CQ) derivatives (**D01**–**D21**) were evaluated for their anti-MTB activity; among them nine (**D01**–**D09**) showed MIC values between 7.3 and 142 µM. Additionally, **D01**, **D04**, and **D05** did not show a significant cytotoxic profile in the lowest concentration evaluated by the Mosmann’s MTT assay. Moreover, an HQSAR analysis was performed using these nine compounds and 33 more 7CQ derivatives, considering the amino (I) and imino (II) tautomers as different data sets, resulting in two robust models with good correlation and predictive capability for the anti-MTB activity.

The model obtained considering the 4-imino-quinoline scaffold (tautomer II) showed significant statistical values (q^2^= 0.77; r^2^= 0.98; SE= 0.10 SE_cv_= 0.35), besides, its HQSAR contribution map can explain the importance of structural fragments to the overall activity of the polysubstituted derivatives. Among the fragments, hydrazine moiety and two bulky substituents in the phenyl ring, such as the methoxy group, are important for improving the biological activity. Among the compounds with poor solubility (**D10**–**D21**), the HQSAR model II predicted high biological activity for **D21**, for this reason, thermodynamic and kinetic studies could be made to determine the dissolution rates to assess its biological activity.

## Figures and Tables

**Table 1:**
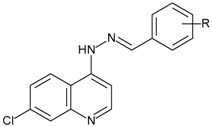
Chemical structures (R substituent), anti-mycobacterial (minimum inhibitory concentration, MIC) and cytotoxicity (cell viability at drug doses of 2.5, 25, and 100 μg/mL) activities of series **D** (**D01**–**D21**) and ethambutol (EMB).

#	R substituent	MIC (μM) ^a,b^	Cell Viability ^c,d^ (%)/Dose (µg/mL)		
			2.5	25	100
**D01**	2,6-Cl	142.6	100	90	86
**D02**	2,3-OH	79.7	83	40	83
**D03**	3,4-OH	318.7	78	35	72
**D04**	2,5-OH	79.7	100	100	70
**D05**	2,3-OCH_3_	7.3	98	97	85
**D06**	3,4-OCH_3_	7.3	65	8	20
**D07**	2,5-OCH_3_	18.3	40	22	47
**D08**	3,4,5-OCH_3_	16.8	93	90	69
**D09**	2-Cl, 3-OH, 4-OCH_3_	17.3	80	55	48
**D10**	2,3-Cl	n.d.	n.d.	n.d.	n.d.
**D11**	2,4-Cl	n.d.	n.d.	n.d.	n.d.
**D12**	3,4-Cl	n.d.	n.d.	n.d.	n.d.
**D13**	2,4-OH	n.d.	n.d.	n.d.	n.d.
**D14**	2,3,4-OH	n.d.	n.d.	n.d.	n.d.
**D15**	3,4,5-OH	n.d.	n.d.	n.d.	n.d.
**D16**	2,4-OCH_3_	n.d.	n.d.	n.d.	n.d.
**D17**	2-OH, 4-CH_3_	n.d.	n.d.	n.d.	n.d.
**D18**	2-OH, 5-CH_3_	n.d.	n.d.	n.d.	n.d.
**D19**	3-Cl, 4-OH	n.d.	n.d.	n.d.	n.d.
**D20**	2-OH, 3-OCH_3_	n.d.	n.d.	n.d.	n.d.
**D21**	3-NO_2_, 4-Cl	n.d.	n.d.	n.d.	n.d.
**EMB**	----	15.3	100	93	82

^a^ Anti-mycobacterial activity (minimum inhibitory concentration, MIC) against the *Mycobacterium tuberculosis* wild-type H37Rv strain susceptible to streptomycin, isoniazid, rifampin, and ethambutol (S.I.R.E.) drugs (American Type Culture Collection, ATCC^®^ 27294™) by the microplate Alamar blue assay (MABA); ^b^ Not determined (n.d.) because the compound was insoluble in the culture medium; ^c^ Cell viability for macrophage cell line J774 (ATCC^®^ TIB-67™) by the Mosmann’s MTT assay.

**Table 2:**
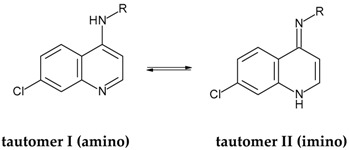
Chemical structures (tautomers I and II, R substituent) and biological activity values (pMIC) of the 7-chloro-4-aminoquinoline derivatives for series **A**‒**D**.

# ^a^	R substituent	pMIC ^b^	# ^a^	R substituent	pMIC ^b^	# ^a^	R substituent	pMIC ^b^
A01*	-(CH_2_)_2_Cl	4.29	B02*	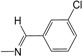	5.01	**C03**	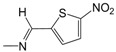	5.43
**A02**	-(CH_2_)_2_N_3_	3.69	**B03**	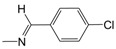	4.40	**C04**	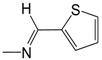	4.96
A03*	-(CH_2_)_2_-NH-(CH_2_)_2_CH_3_	3.42	**B04**	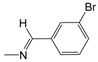	5.06	**C05**	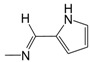	4.94
**A04**	-(CH_2_)_2_-NH-C_6_H_11_	3.48	B05*	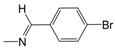	5.16	**C06**	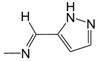	4.94
**A05**	-(CH_2_)_2_-NH-Ph	3.77	**B06**	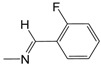	4.98	**C07**	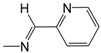	4.35
**A06**	-(CH_2_)_6_-NH_2_	4.05	**B07**	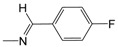	5.08	D01*	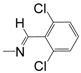	3.85
A07*	-(CH_2_)_8_-NH_2_	4.69	**B08**	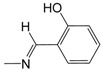	5.08	**D02**	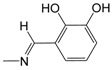	4.10
**A08**	-(CH_2_)_10_-NH_2_	5.03	**B09**	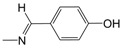	4.68	**D03**	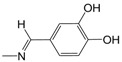	3.50
**A09**	-CH_3_	3.28	**B10**	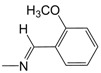	5.00	D04*	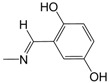	4.10
**A10**	-(CH_2_)_2_CH_3_	3.64	**B11**	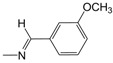	5.00	**D05**	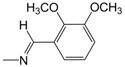	5.14
A11*	-CH_2_(CH_3_)_2_	3.64	**B12**	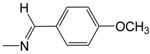	5.10	**D06**	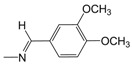	5.14
**A12**	-(CH_2_)_3_CH_3_	4.27	**B13**	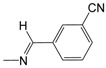	4.69	**D07**	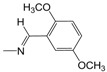	4.74
**A13**	-(CH_2_)_3_Cl	3.71	**C01**	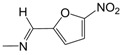	5.10	**D08**	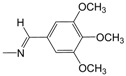	4.77
**B01**	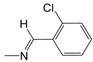	4.70	C02*	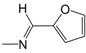	4.94	D09*	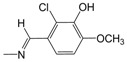	4.76

^a^ The test set compounds from series **A**, **B**, **C**, and **D** are marked with an asterisk (*). ^b^ pMIC (M) = –Log of the minimum inhibitory concentration (MIC) on *Mycobacterium tuberculosis*.

**Table 3:** Summary of hologram quantitative structure–activity relationship (HQSAR) statistical indexes for various fragment distinction (FD) parameters using the default fragment size (FS = 4–7 atoms) for both tautomers I (amino) and II (imino) data sets.

FD ^b^	Tautomer I/Statistical Indexes ^a^						Tautomer II/Statistical Indexes ^a^					
	q^2^	r^2^	SE	SE_cv_	NC	HL	q^2^	r^2^	SE	SE_cv_	NC	HL
**A/B**	0.63	1.00	0.05	0.52	15	83	0.66	0.97	0.13	0.42	9	61
**A/C**	**0.80**	**0.97**	**0.12**	**0.32**	**8**	**53**	**0.77**	**0.98**	**0.10**	**0.35**	**10**	**53**
**A/H**	0.58	0.91	0.21	0.45	7	59	0.58	0.91	0.21	0.45	7	59
**A/Ch**	0.70	1.00	0.05	0.44	13	401	0.68	1.00	0.05	0.45	13	401
**A/DA**	0.61	1.00	0.06	0.53	15	307	0.61	1.00	0.06	0.53	15	307
**B/C**	0.69	0.97	0.15	0.47	15	59	0.68	0.96	0.15	0.44	12	59
**B/H**	0.33	0.88	0.25	0.59	9	307	0.44	0.90	0.23	0.55	9	307
**C/H**	0.54	0.97	0.15	0.58	15	257	0.61	0.97	0.14	0.50	13	199
**C/DA**	0.77	0.99	0.09	0.39	14	97	0.72	0.99	0.08	0.45	15	83
**A/B/C**	0.62	1.00	0.04	0.52	15	401	0.68	0.99	0.08	0.42	10	257
**A/C/H**	0.79	0.98	0.10	0.33	9	83	0.68	1.00	0.03	0.48	15	199
**A/B/C/H**	0.67	1.00	0.02	0.49	15	307	0.73	1.00	0.01	0.44	15	307

^a^ q^2^: leave-one-out (LOO) cross-validated r^2^; r^2^: squared correlation coefficient; SE_cv_: cross-validated standard error; SE: standard error; NC: optimal number of components; HL: hologram length; ^b^ Fragment distinction parameters: atoms (A), bonds (B), connections (C), chirality (Ch), hydrogen (H) atoms, and donor/acceptor (DA) atoms.

**Table 4:** Summary of hologram quantitative structure–activity relationship (HQSAR) statistical indexes for the influence of various fragment sizes (FS) using the fragment distinction (FD) parameters atoms and connections (A/C) for both tautomers I (amino) and II (imino) data sets.

FS	Tautomer I/Statistical Indexes ^a^						Tautomer II/Statistical Indexes ^a^					
	q^2^	r^2^	SE	SE_cv_	NC	HL	q^2^	r^2^	SE	SE_cv_	NC	HL
**2‒5**	0.61	0.99	0.09	0.53	15	257	0.70	0.99	0.08	0.47	15	257
**3‒6**	0.61	0.99	0.07	0.52	14	353	0.68	0.99	0.07	0.47	14	151
**4‒7**	**0.80**	**0.97**	**0.12**	**0.32**	**8**	**53**	**0.77**	**0.98**	**0.10**	**0.35**	**10**	**53**
**5‒8**	0.63	1.00	0.04	0.48	12	353	0.75	1.00	0.03	0.43	15	61
**6‒9**	0.64	0.96	0.14	0.41	6	353	0.67	0.98	0.10	0.40	7	257
**7‒10**	0.66	1.00	0.01	0.49	15	257	0.62	1.00	0.02	0.50	13	257
**8‒11**	0.60	0.98	0.09	0.46	9	97	0.57	1.00	0.01	0.56	15	257
**9‒12**	0.53	0.98	0.10	0.50	9	307	0.57	0.99	0.07	0.49	10	353

^a^ q^2^: leave-one-out (LOO) cross-validated r^2^; r^2^: squared correlation coefficient; SE_cv_: cross-validated standard error; SE: standard error; NC: optimal number of components; HL: hologram length.

**Table 5:** Values of R, q^2^, and r^2^ of ten HQSAR runs generated for each randomized dataset (tautomers I and II, *N* = 32) using the Y-randomization test.

Randomization	R ^a^	Tautomer I Statistical Indexes ^b^		Tautomer II Statistical Indexes ^b^	
		q^2^	r^2^	q^2^	r^2^
**1**	−0.20	0.107	0.458	0.078	0.267
**2**	−0.21	0.178	0.301	0.138	0.526
**3**	−0.43	0.212	0.726	0.410	0.632
**4**	−0.11	0.298	0.565	0.172	0.688
**5**	−0.02	n.p.	n.p.	n.p.	n.p.
**6**	0.07	n.p.	n.p.	n.p.	n.p.
**7**	−0.03	n.p.	n.p.	n.p.	n.p.
**8**	−0.11	n.p.	n.p.	n.p.	n.p.
**9**	−0.06	n.p.	n.p.	n.p.	n.p.
**10**	0.02	n.p.	n.p.	n.p.	n.p.

^a^ R: simple correlation coefficient between the random and original orders of the experimental pMIC values. ^b^ q^2^: leave-one-out (LOO) cross-validated r^2^; r^2^: squared correlation coefficient; n.p.: no predictive model.

**Table 6:** Experimental pMIC (pMIC_Exp_), calculated pMIC (pMIC_Calc_), and residual (Res = pMIC_Exp_ − pMIC_Calc_) values of the tautomers I (amino) and II (imino) data sets using the best HQSAR models.

# ^a^	pMIC_Exp_^b^	Tautomer I		Tautomer II		#^a^	pMIC_Exp_^b^	Tautomer I		Tautomer II	
		pMIC_Calc_	Res ^c^	pMIC_Calc_	Res ^c^			pMIC_Calc_	Res ^c^	pMIC_Calc_	Res ^c^
A01*	4.29	3.57	**0.72**	3.61	**0.68**	**B09**	4.68	4.55	0.13	4.68	0.00
**A02**	3.69	3.64	0.06	3.73	−0.04	**B10**	5.00	5.14	−0.14	5.07	−0.07
A03*	3.42	3.44	−0.02	3.68	−0.26	**B11**	5.00	4.97	0.03	5.06	−0.06
**A04**	3.48	3.42	0.06	3.42	0.06	**B12**	5.10	5.07	0.02	5.21	−0.12
**A05**	3.77	3.81	−0.04	3.82	−0.04	**B13**	4.69	4.63	0.07	4.68	0.01
**A06**	4.05	4.25	−0.20	4.20	−0.16	**C01**	5.10	5.05	0.05	5.13	−0.03
A07*	4.69	4.68	0.01	4.61	0.08	C02*	4.94	4.21	**0.73**	4.59	0.35
**A08**	5.03	5.10	−0.07	5.02	0.01	**C03**	5.43	5.45	−0.02	5.46	−0.03
**A09**	3.28	3.26	0.03	3.28	0.00	**C04**	4.96	4.91	0.05	4.98	−0.02
**A10**	3.64	3.74	−0.09	3.80	−0.16	**C05**	4.94	4.98	−0.04	4.91	0.03
A11*	3.64	3.68	−0.04	3.93	−0.29	**C06**	4.94	5.03	−0.09	4.90	0.04
**A12**	4.27	4.01	0.27	4.01	0.26	**C07**	4.35	4.32	0.04	4.27	0.08
**A13**	3.71	3.73	−0.02	3.67	0.04	D01*	3.85	4.44	**−0.60**	4.21	−0.37
**B01**	4.70	4.69	0.01	4.65	0.06	**D02**	4.10	4.15	−0.05	4.07	0.03
B02*	5.01	4.46	**0.55**	4.64	0.36	**D03**	3.50	3.74	−0.24	3.63	−0.13
**B03**	4.40	4.52	−0.12	4.47	−0.07	D04*	4.10	4.04	0.06	4.03	0.06
**B04**	5.06	5.15	−0.09	5.07	−0.01	**D05**	5.14	5.19	−0.05	5.20	−0.07
B05*	5.16	5.31	−0.15	5.02	0.14	**D06**	5.14	4.96	0.18	5.06	0.07
**B06**	4.98	4.93	0.06	4.98	0.01	**D07**	4.74	4.74	0.00	4.66	0.08
**B07**	5.08	5.09	−0.01	5.04	0.04	**D08**	4.77	4.78	0.00	4.74	0.03
**B08**	5.08	4.87	0.20	4.93	0.15	D09*	4.76	4.32	0.44	4.24	**0.53**

^a^ Test set compounds are marked with an asterisk (*). ^b^ pMIC (M): −Log of the minimum inhibitory concentration (MIC). ^c^ The residual values in bold correspond to outliers that have been defined as any compound showing residual values higher than twice the standard deviation (SD) of the estimate of the model (tautomer I dataset: 2 * SD = 0.47; tautomer II dataset: 2 * SD = 0.38).

**Table 7:** Calculated pMIC (pMIC_Calc_) values for **D10**–**D21** of tautomers II (R substituent) using the A/C (atoms and connections) HQSAR model.

#	R substituent	pMIC_Calc_	#	R substituent	pMIC_Calc_
**D10**	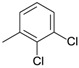	4.32	**D16**	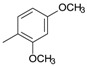	5.07
**D11**	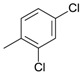	4.00	**D17**	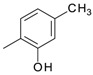	4.56
**D12**	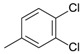	4.15	**D18**	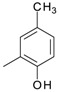	4.97
**D13**	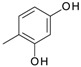	4.50	**D19**	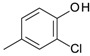	4.19
**D14**	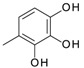	3.09	**D20**	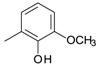	4.80
**D15**	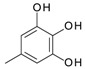	2.58	**D21**	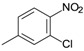	5.97

## References

[1] World Health Organization (2015). Global Tuberculosis Report 2015.

[2] Welsch M.E., Snyder S.A., Stockwell B.R. (2010). Privileged scaffolds for library design and drug discovery. Curr. Opin. Chem. Biol..

[3] Montenegro R.C., Lotufo L.V., de Moraes M.O., do O Pessoa C., Rodrigues F.A.R., Bispo M.L.F., Freire B.A., Kaiser C.R., de Souza M.V.N. (2012). Cytotoxic activity of polysubstituted 7-chloro-4-quinolinyl-hydrazone derivatives. Lett. Drug Des. Discov..

[4] Mitton-Fry M.J., Brickner S.J., Hamel J.C., Brennan L., Casavant J.M., Chen M., Chen T., Ding X., Driscoll J., Hardink J. (2013). Novel quinoline derivatives as inhibitors of bacterial DNA gyrase and topoisomerase IV. Bioorg. Med. Chem. Lett..

[5] Casagrande M., Barteselli A., Basilico N., Parapini S., Taramelli D., Sparatore A. (2012). Synthesis and antiplasmodial activity of new heteroaryl derivatives of 7-chloro-4-aminoquinoline. Bioorg. Med. Chem..

[6] Coimbra E.S., Antinarelli L.M.R., da Silva A.D., Bispo M.L.F., Kaiser C.R., de Souza M.V.N. (2013). 7-Chloro-4-quinolinyl hydrazones: a promising and potent class of antileishmanial compounds. Chem. Biol. Drug Des..

[7] Sun X.H., Guan J.Q., Tan J.J., Liu C., Wang C.X. (2012). 3D-QSAR studies of quinoline ring derivatives as HIV-1 integrase inhibitors. SAR QSAR Environ. Res..

[8] Palomino J.C., Martin A. (2013). TMC207 becomes bedaquiline, a new anti-TB drug. Future Microbiol..

[9] Andries K. (2005). A diarylquinoline drug active on the ATP synthase of *Mycobacterium tuberculosis*. Science.

[10] Candéa A.L.P., Ferreira M.L., Pais K.C., Cardoso L.N.F., Kaiser C.R., Henriques M.G.M.O., Lourenço M.C.S., Bezerra F.A.F.M., de Souza M.V.N. (2009). Synthesis and antitubercular activity of 7-chloro-4-quinolinylhydrazones derivatives. Bioorg. Med. Chem. Lett..

[11] De Souza M.V.N., Pais K.C., Kaiser C.R., Peralta M.A., Ferreira M.L., Lourenço M.C.S. (2009). Synthesis and in vitro antitubercular activity of a series of quinoline derivatives. Bioorg. Med. Chem..

[12] Ferreira M.L., Gonçalves R.S., Cardoso L.N.F., Kaiser C.R., Candéa A.L.P., Henriques M.G.M.O., Lourenço M.C.S., Bezerra F.A.F.M., de Souza M.V.N. (2010). Synthesis and antitubercular activity of heteroaromatic isonicotinoyl and 7-chloro-4-quinolinyl hydrazone derivatives. Sci. World J..

[13] Gonçalves R.S., Kaiser C.R., Lourenço M.C., Bezerra F.A., de Souza M.V.N., Wardell J.L., Wardell S.M., Henriques M.G.M.O., Costa T. (2012). Mefloquine–oxazolidine derivatives, derived from mefloquine and arenecarbaldehydes: in vitro activity including against the multidrug-resistant tuberculosis strain T113. Bioorg. Med. Chem..

[14] Lima C.H.S., de Alencastro R.B., Kaiser C.R., de Souza M.V.N., Rodrigues C.R., Albuquerque M.G. (2015). Aqueous molecular dynamics simulations of the *M. tuberculosis* enoyl-ACP reductase-NADH system and its complex with a substrate mimic or diphenyl ethers inhibitors. Int. J. Mol. Sci..

[15] Vergara F.M.F., Lima C.H.S., Henriques M.G.M.O., Candéa A.L.P., Lourenço M.C.S., Ferreira M.L., Kaiser C.R., de Souza M.V.N. (2009). Synthesis and antimycobacterial activity of *N′*-[(*E*)-(monosubstituted-benzylidene)]-2-pyrazinecarbohydrazide derivatives. Eur. J. Med. Chem..

[16] Heritage T.W., Lowis D.R., Parrill A.L., Reddy M.R. (1999). Molecular Hologram QSAR. Rational Drug Design.

[17] de Souza S.D., de Souza A.M., de Sousa A.C., Sodero A.C., Cabral L.M., Albuquerque M.G., Castro H.C., Rodrigues C.R. (2012). Hologram QSAR models of 4-[(diethylamino)methyl]-phenol inhibitors of acetyl/butyrylcholinesterase enzymes as potential anti-Alzheimer agents. Molecules.

[18] Leal F.D., da Silva Lima C.H., de Alencastro R.B., Castro H.C., Rodrigues C.R., Albuquerque M.G. (2015). Hologram QSAR models of a series of 6-arylquinazolin-4-amine inhibitors of a new Alzheimer’s disease target: dual specificity tyrosine-phosphorylation-regulated kinase-1A enzyme. Int. J. Mol. Sci..

[19] Rodrigues C.R., Flaherty T.M., Springer C., McKerrow J.H., Cohen F.E. (2002). CoMFA and HQSAR of acylhydrazide cruzain inhibitors. Bioorg. Med. Chem. Lett..

[20] Myint K.Z., Xie X.-Q. (2010). Recent advances in fragment-based QSAR and multi-dimensional QSAR methods. Int. J. Mol. Sci..

[21] Franzblau S.G., Witzig R.S., McLaughlin J.C., Torres P., Madico G., Hernandez A., Degnan M.T., Cook M.B., Quenzer V.K., Ferguson R.M., Gilman R.H. (1998). Rapid, low-technology MIC determination with clinical *Mycobacterium tuberculosis* isolates by using the microplate Alamar Blue assay. J. Clin. Microbiol..

[22] Canetti G., Rist N., Grosset J. (1963). Measurement of sensitivity of the tuberculous bacillus to antibacillary drugs by the method of proportions. Methodology, resistance criteria, results and interpretation. Rev. Tuberc. Pneumol..

[23] Bhattachar S.N., Deschenes L.A., Wesley J.A. (2006). Solubility: it’s not just for physical chemists. Drug Discov. Today.

[24] Ishikawa M., Hashimoto Y. (2011). Improvement in aqueous solubility in small molecule drug discovery programs by disruption of molecular planarity and symmetry. J. Med. Chem..

[25] Carvalho M.V., Penido C., Siani A.C., Valente L.M.M., Henriques M.G.M.O. (2006). Investigations on the anti-inflammatory and anti-allergic activities of the leaves of *Uncaria guianensis* (Aublet) J. F. Gmelin. Inflammopharmacology.

[26] Souza M.C., Siani A.C., Ramos M.F.S., Menezes-de-Lima O.J., Henriques M.G.M.O. (2003). Evaluation of anti-inflammatory activity of essential oils from two Asteraceae species. Pharmazie.

[27] Araújo J.Q., de Brito M.A., Hoelz L.V.B., de Alencastro R.B., Castro H.C., Rodrigues C.R., Albuquerque M.G. (2011). Receptor-dependent (RD) 3D-QSAR approach of a series of benzylpiperidine inhibitors of human acetylcholinesterase (HuAChE). Eur. J. Med. Chem..

[28] Rando D.G., Sato D.N., Siqueira L., Malvezzi A., Leite C.Q., do Amaral A.T., Ferreira E.I., Tavares L.C. (2002). Potential tuberculostatic agents. Topliss application on benzoic acid [(5-nitro-thiophen-2-yl)-methylene]-hydrazide series. Bioorg. Med. Chem..

[29] de Souza M.V.N., Bispo M.L.F., Alcantara C.C., Wardell S.M.S.V., Wardell J.L. (2013). Structures of a co-crystal of tautomers of (*E*)-*N*-7-chloro-4-(2-methoxycinnamolyhydrazinyl)quinoline and a single tautomer of the 4-methoxy analogue. Zeitschrift für Krist. Cryst. Mater..

[30] Howie R.A., de Souza M.V.N., Ferreira M.L., Kaiser C.R., Wardell J.L., Wardell S.M.S.V. (2010). Structures of arylaldehyde 7-chloroquinoline-4-hydrazones: supramolecular arrangements derived from N—H···N, C—H···X (X = N, O, or π) and π···π interactions. Zeitschrift für Krist..

[31] Allen F.H. (2002). The Cambridge Structural Database: a quarter of a million crystal structures and rising. Acta Crystallogr. Sect. B Struct. Sci..

[32] Shao Y., Molnar L.F., Jung Y., Kussmann J., Ochsenfeld C., Brown S.T., Gilbert A.T.B., Slipchenko L.V., Levchenko S.V., O’Neill D.P. (2006). Advances in methods and algorithms in a modern quantum chemistry program package. Phys. Chem. Chem. Phys..

[33] Dewar M.J.S., Zoebisch E.G., Healy E.F., Stewart J.J.P. (1985). Development and use of quantum mechanical molecular models. 76. AM1: a new general purpose quantum mechanical molecular model. J. Am. Chem. Soc..

[34] (2008). HQSAR, Sybyl, version 8.0.

[35] Benigni R., Bossa C. (2008). Predictivity of QSAR. J. Chem. Inf. Model..

[36] Fernandes J.P.S., Pasqualoto K.F.M., Ferreira E.I., Brandt C.A. (2011). Molecular modeling and QSAR studies of a set of indole and benzimidazole derivatives as H4 receptor antagonists. J. Mol. Model..

[37] Cramer R.D., Patterson D.E., Bunce J.D. (1988). Comparative molecular field analysis (CoMFA). 1. Effect of shape on binding of steroids to carrier proteins. J. Am. Chem. Soc..

